# Caries of the Teeth
†On Caries of the Teeth, and the Cure of Toothache without Extraction; with explanatory engravings. By Donaldson Mackenzie, Surgeon-Dentist. Churchill. 1855.


**Published:** 1857-04

**Authors:** 


### CARIES OF THE TEETH.†

Mr. Mackenzie argues that caries of the teeth has a common origin which is always *external*, *i.e.*, that it commences *on*, not *in*, the teeth. If a carious tooth be freed by the file from its decayed part, and be kept constantly cleansed, he depends on the cavity being refilled by deposit taking place naturally, in a manner analogous to the porcelain deposit on scraped bone. Speaking of the cure of toothache, the author hazards the

---

† On Caries of the Teeth, and the Cure of Toothache without Extraction; with explanatory engravings. By Donaldson Mackenzie, Surgeon-Dentist. Churchill. 1855.

assertion, "that the guillotine is as legitimate an instrument for the cure of headache, as the forceps for the relief of toothache." Nevertheless, Mr. Mackenzie finds cases where the forceps cannot be dispensed with. Looking on toothache as inflammation of the dental pulp, he treats it by general measures, and the local and frequent application of anodynes. For cleansing the teeth, he recommends washes rather than powders.

